# Participating in the digital world: a consensus statement on digital social contact for people with disabilities living in sheltered care facility homes

**DOI:** 10.1080/20473869.2023.2190115

**Published:** 2023-03-24

**Authors:** Linda N. Douma, Anne Tharner, Paula S. Sterkenburg, Lotte Piekema, Annet ten Brug, Noud Frielink, Lianne Bakkum, Esmee Adam, J. Clasien de Schipper, Petri Embregts, Carlo Schuengel

**Affiliations:** 1 Department of Clinical Child and Family Studies, Amsterdam Public Health Research Institute, Vrije Universiteit Amsterdam, Amsterdam, The Netherlands; 2 Viveon, Amsterdam, The Netherlands; 3 Bartiméus, Doorn, The Netherlands; 4 Department of Inclusive and Special Needs Education, University of Groningen, Groningen, The Netherlands; 5 Academic Collaborative Centre PMID, Groningen, The Netherlands; 6 Tranzo, Tilburg School of Social and Behavioural Sciences, Tilburg University, Tilburg, The Netherlands

**Keywords:** consensus statement, digital social contact, intellectual disabilities, multiple disabilities, participation

## Abstract

**Introduction:**

Digital social contact is increasingly being used, which accelerated during the COVID-19 pandemic. This study aimed to determine the consensus among stakeholders regarding recommendations for the use and facilitation of digital social contact for people with intellectual disabilities living in sheltered care facility homes.

**Methods:**

This consensus statement was developed in three consecutive rounds of questionnaires (rapid online modified Delphi design). The expert-groups included people with disabilities (*N* = 6) and their families (*N* = 10), support professionals (*N* = 9), behavioural consultants (*N* = 7), managers of sheltered care facility homes (*N* = 10), scientists and industry experts (*N* = 15).

**Findings:**

Four main themes were identified: 1. Reasons for and types of digital social contact; 2. Support and training needs; 3. Materials and other requirements needed to enable digital social contact; and 4. Best practices and future developments. For each theme, several recommendations were formulated.

**Discussion and conclusion:**

This study resulted in a consensus statement aimed mainly at care professionals, families of people with intellectual disabilities and managers of sheltered care facility homes. Findings show that digital social contact can contribute to societal participation of people with disabilities. Additionally, tailored exploration of digital contact is recommended, as well as aiming for inclusive-by-design technology developments with developers and stakeholders working together.

## Introduction

Digital forms of social contact – such as emailing, texting, video calling and social media – have increasingly become a part of people’s daily life. People with intellectual disabilities and their social networks could benefit from participating in this digital world (Goggin *et al.* 2017). In this present study, we aimed to integrate input from scientific literature and practice on digital participation, resulting in a consensus statement about the use of digital forms of social contact by people with intellectual disabilities living in sheltered care facility homes. It should be mentioned that we acknowledge the importance of in-person contact and are not advocating using digital social contact as a long-term alternative for it (Courtenay and Perera 2020, Embregts *et al.* 2022).

The use of digital forms of social contact further accelerated because of social distancing measures during the COVID-19 pandemic (Nguyen *et al.* 2020, Boeije *et al.* 2021, Chadwick *et al.* 2022). Restrictions on in-person contact implemented worldwide to limit the spread of SARS-CoV-2 were particularly strict for people with intellectual disabilities living in sheltered care facility homes (inclusive of extra-care homes) – such as facility homes for people with mild or moderate intellectual disabilities and people with profound intellectual and multiple disabilities – to protect this potentially vulnerable group (Comas-Herrera *et al.* 2021). In addition, these restrictions were exceptionally impactful for this group of people, as they are more likely to have small social networks and less likely to form meaningful relationships and be socially included in comparison to people without disabilities (Van Asselt-Goverts *et al.* 2015, Simões and Santos 2016). In this situation of urgent need for alternatives for social contact, the heterogeneous group of people with intellectual disabilities living in sheltered care facility homes and their social networks – just like many people without disabilities – started to explore digital means for social interaction. This led to new insights into potential benefits and downsides as well as requirements regarding digital forms of social contact used by people with intellectual disabilities living in sheltered care facility homes (Araten-Bergman and Shpigelman 2021, Boeije *et al.* 2021, Embregts *et al.* 2022, Honingh *et al.* 2021, Bakkum *et al.* 2022).

Our recent review of the scientific literature reported that, prior to the COVID-19 pandemic, limited research has been done on whether digital forms of social contact meet the needs of people with intellectual disabilities living in sheltered care facility homes and their families and friends (Bakkum *et al.* 2021). Thus, at the beginning of the COVID-19 pandemic there was not much scientific knowledge about the possible benefits, downsides and requirements regarding digital social contact, particularly within a context of in-person contact restrictions. The little research that had been conducted, indicated that, generally, digital social contact has the potential of being beneficial for the well-being of people with intellectual disabilities living in sheltered care facility homes. It allows them to stay connected with other people (Lancioni *et al.* 2013, Ramsten *et al.* 2019, Shpigelman 2018, Jutai and Tuazon 2022, Spassiani *et al.* 2022), perhaps particularly when used in addition to in-person contact (Araten-Bergman and Shpigelman 2021, Dyzel *et al.* 2020).

Therefore, digital social contact might also be beneficial to explore for those who are not engaging in digital contact – mainly, but not limited to, people with severe multiple disabilities – as it can contribute to their inclusion and participation (Hanzen *et al.* 2017). However, to engage in meaningful digital social contact, there are also potential barriers (e.g. practical or literacy difficulties) (Shpigelman 2018, Ramsten *et al.* 2020) and downsides (e.g. experiencing stress and frustration) (Araten-Bergman and Shpigelman 2021, Ramsten *et al.* 2020, Shpigelman and Gill 2014, Honingh *et al.* 2021) as well as requirements (e.g. cultural, technical, support) (Lancioni *et al.* 2013, Ramsten *et al.* 2019, Frielink *et al.* 2021, Parsons *et al.* 2008, Honingh *et al.* 2021) to consider. In general, the use of digital devices and applications could be made easier for people with disabilities, fostering broader, more effective and independent use (Dyzel *et al.* 2020, Ramsten *et al.* 2020, Goggin *et al.* 2017).

The aim of the current study was to determine consensus concerning the use and facilitation of digital social contact for people with intellectual disabilities in sheltered care facility homes, building on findings from literature and recent practice, using a Delphi study approach (Murphy *et al.* 1998, Trevelyan and Robinson 2015). The findings of our study can be used as a guide for sheltered care facility homes and may contribute to meaningful participation and inclusion of people with intellectual disabilities in a digital society, as supported by other studies (Araten-Bergman and Shpigelman 2021, Embregts *et al.* 2022, Goggin *et al.* 2017, Barlott *et al.* 2020, Alfredsson Ågren *et al.* 2020).

Specifically, the main research question addressed in this study was:

1. What is the consensus among stakeholders (e.g. people with intellectual disabilities and their families, support professionals and management) regarding overarching recommendations and considerations for the use and facilitation of digital social contact for people with intellectual disabilities living in sheltered care facility homes?

The following subquestions were also addressed:

- Are there differences in recommendations and considerations for people with mild or moderate intellectual disabilities and people with profound intellectual and multiple disabilities?
- Who would mostly make use of information and advice on digital social contact, and/or who is responsible for acting on the recommendations?

## Methods of consensus development

### Study design

This consensus statement was developed in three consecutive rounds over a period of five weeks (April 19–May 17 2021), following a rapid online modified Delphi design (Silva *et al.* 2018, Hasson *et al.* 2000). A *Delphi* design was chosen as a method to reach consensus because it allows for the inclusion of a heterogeneous group of with a wide range of knowledge and experiences. Moreover, as conducted without interactions among participants, group dynamics are prevented from inadvertently reducing the impact of minority positions (Murphy *et al.* 1998, Trevelyan and Robinson 2015). To achieve this, the current study used an *online survey* format with mostly *closed-ended questions* (Winkler and Moser 2016, Trevelyan and Robinson 2015). Additionally, as we previously conducted a systematic review study on the subject (Bakkum *et al.* 2021) as well as a survey and interview study collecting a wide range of experiences (Bakkum *et al.* 2022)*(under review)*, a Delphi study would allow for a synthesis of these findings from literature and practice, resulting in a consensus statement to be used as a guide for sheltered care facility homes (Murphy *et al.* 1998, Trevelyan and Robinson 2015). A *rapid* online modified Delphi design was chosen because of two main reasons: 1) the need for swift results considering the ongoing COVID-19 context; and 2) we were able to use findings from the two studies we previously conducted (as mentioned above), which formed the basis for developing the questions for the first two Delphi rounds.

### Participants

Our research group is a collaboration between four Academic Collaborative Centres across the Netherlands, that structurally work together with health care organisations and sheltered care facility homes, with a research focus on the care for and well-being of people with mild or moderate intellectual disabilities and people with profound intellectual and multiple disabilities. Using the network of our research group and associated institutes across the Netherlands, applying a combination of targeted and convenience sampling, we recruited a group of stakeholders/experts (*N* = 57) to participate in our Delphi study. Every round, we invited all 57 recruited participants to contribute. However, not all of them participated in each round. Round one had 49 participants, round two had 50 and round three had 38. Participants gave written informed consent for participation. Using an a-priori defined recruitment frame, we aimed for diversity in experts by actively recruiting them from the following populations: 1) People with mild intellectual disabilities (some also had a visual impairment), who were trained to use their experiences to advocate for themselves and other people with intellectual disabilities (i.e. experts-by-experience) (*N* = 6); 2) Family members of people with disabilities, practising as self-advocates (*N* = 10); 3) Support professionals (*N* = 9); 4) Behavioural consultants (*N* = 7); 5) Managers and directors of sheltered care facility homes (*N* = 10); 6) Scientists and industry experts in the field of disability care and/or health technology (*N* = 15). Participants knew which other expert-groups were participating, but did not know who the other participants were. Additionally, we made sure we included both participants who had expertise regarding care for people with mild or moderate intellectual disabilities and participants who had expertise regarding care for people with profound intellectual and multiple disabilities. The common factor was that it always concerned care for people living in sheltered care facility homes. Furthermore, part of the recruitment frame was a predetermined matrix consisting of a variety of combinations regarding gender, age (18+), Dutch region and health care organisation was used as a flexible guide to ensure diversity in these variables. To limit the collection of personal data, we only used aggregated levels of characteristics (e.g. age group instead of birth date) for recruitment. During the period that we conducted this study, people living in sheltered care facility homes in the Netherlands were allowed in-person contact with one to two persons from outside their facility home per day. This study received ethical approval of the Scientific and Ethical Review Board (VCWE) of the Faculty of Behaviour & Movement Sciences, Vrije Universiteit Amsterdam (VCWE-2021-034R1).

### Administration of the questionnaires

Each Delphi round consisted of an online questionnaire, using mostly closed-ended questions and some open-ended questions. The first round also contained one ranking question. The online questionnaires were distributed via personal email using an anonymous link, meaning we do not know which answers belong to which individual or expert-group. This anonymity was in accordance with our aim to weigh the answers of all participants as equally important and to generate a consensus statement that represented the group of experts as a whole. Participants were allowed to skip questions. Based on a pre-test and the software used to design the online questionnaires, we expected that the first round would take participants five to fifteen minutes to fill in, and that the second and third rounds would take them ten to thirty minutes. The online survey data collected on duration time confirmed these expectations. Participants had one week to fill in each questionnaire.

### Development of the questionnaires

A qualitative descriptive analysis of the findings from our systematic review (Bakkum *et al.* 2021) and survey and interview study (Bakkum *et al.* 2022) (as aforementioned) as well as other recent publications on digital social contact (e.g. Araten-Bergman and Shpigelman 2021, Frielink *et al.* 2021) guided the development of the questions used in this Delphi study, especially regarding the first and second Delphi rounds. The findings of these previous studies showed that in the context of digital social contact themes such as well-being, staying connected, benefits and downsides, requirements, exploration and learning, support, privacy and participation were important. Findings also showed that people with a variety of disabilities were participating in digital social contact. the first and second Delphi rounds were used to develop the next round. After each round, we assessed whether consensus was reached on the closed-ended questions. If consensus was not reached, questions were reformulated for the next round. For a more detailed description, see below. In the second and third rounds, participants received general feedback on the results of the previous round as a prelude to the follow-up questions that were to be asked in this particular round. For example, participants received the feedback that ‘most of the participants who filled in the previous questionnaire believed that […]’ or ‘the participants who filled in the previous questionnaire differed in their opinion on […]’. The designing of each round and interpreting of the results involved extensive discussions by our research group. We used several means to assure that our participants with intellectual disabilities were able to understand and fill in the questionnaires, such as easy-to-read language, starting each sentence on a new line, using a ranking or Likert-scale with smileys, the use of pictures and the allowance of some support with filling in the questionnaires. Furthermore, when constructing the questionnaires, we received input and advice from several experts-by-experience (not participating in our study) on comprehension of the questionnaires and ease of completion either independently or with support from a family member or direct support professional.

### Analyses of the questionnaires

After each round, we assessed whether consensus was reached (i.e. percentage of participants that gave the same answer) regarding the closed-ended questions (excluding the ranking question in round one), using a 70% agreement score as a cut-off point (Keeney *et al.* 2006, Silva *et al.* 2018). For the ranking question, we calculated a mean ranking score and mode. A thematic analysis was performed on the responses to all open-ended questions presented in the three Delphi rounds, using Atlas.ti version 9.0 (Braun and Clarke 2006). The steps of open coding (marking relevant keywords in the raw data), axial coding (clustering the keyword-codes into group-codes) and selective coding (deriving overarching themes) were followed (Strauss and Corbin 1990), with the topics mentioned in round one (including innovation) acting as sensitising concepts to depart from in studying the data (Bowen 2006). This thematic analysis added context-information for a next Delphi round as well as for the final results part of this consensus statement (see Table 1).

### First round

The first round focussed on assessing which areas in relation to ‘digital social contact between people with disabilities living in sheltered care facility homes and their social networks’ were most relevant to consider and provide information about in a future guideline. We assessed four main areas that had emerged from previous studies of ourselves and others (e.g. Bakkum *et al.* 2021, Araten-Bergman and Shpigelman 2021, Ramsten *et al.* 2020, Frielink *et al.* 2021) to be mainly relevant for this topic: A) Reasons for and types of digital social contact; B) Materials and other requirements needed to enable digital social contact; C) Service user support needs and possibilities; and D) Do’s and don’ts for organising and using digital social contact in sheltered care facility homes. We presented participants with these areas and asked them to prioritise the areas (single items) on a scale of one *(least important)* to four *(most important*). A mean ranking score (range 1-4) was calculated for each area based on ranking placement and the number of people ranking the option in the same place. Following the introduction of these four main areas, participants were invited to raise additional areas that they believed were also relevant (open-ended question). Based on the responses on this question, a fifth area was introduced in the following rounds, namely ‘innovation’. In addition, participants were asked which group of people, in the setting of sheltered care facility homes, would mostly make use of information and advice on digital social contact. Answer options (multiple choice) were: service users; families of people with disabilities; support professionals; managers; directors; specialists, like physicians or behavioural consultants; IT professionals; technical services; I do not know; otherwise.

### Second round

After calculation of consensus as described above, discussion of the findings from round one and considering findings from previous studies, we formulated a set of overarching recommendations within each of the now five main areas for the second round. Participants were asked to indicate for each recommendation whether they would recommend it or not (*I recommend it – either way is fine – I do not recommend it – I do not know*). Participants could choose for which target group (relating to their experience/expertise) they wanted to answer the questions: a) People with mild or moderate intellectual disabilities (*N* = 21); b) People with profound intellectual and multiple disabilities (*N* = 11); and c) Both target groups (*N* = 18). We first presented participants with the recommendations belonging to the main area ranked as most important in round one, and ended with the recommendations belonging to the newly introduced area ‘innovation’. For area A, we formulated seven recommendations (e.g. ‘digital social contact can be used as a way for people with disabilities to participate in society’, ‘digital social contact can be used as a way to stay connected with each other’). Area B comprised seven recommendations (e.g. ‘use standard programmes like WhatsApp and FaceTime for digital social contact’, ‘use specialised programmes of health care organisations for digital social contact’). Area C comprised eight recommendations (e.g. ‘the possibility of using digital social contact should be part of standard care for people with disabilities’, ‘health care organisations should provide people with disabilities with some form of digital skills training’). Area D, ranked as least important area, comprised one recommendation (i.e. ‘support professionals should talk more with each other about the possibilities of digital social contact (i.e. share best practices)’). Lastly, the area ‘innovation’, comprised five recommendations (e.g. ‘develop new programmes and technologies for digital social contact for people with disabilities’, ‘make the current appliances and programmes for digital social contact easier to use for people with disabilities’). Table 1 incorporates all the recommendations that participants were presented with and reached a final consensus about. In round two we also asked participants to share what they believed to be the most important advice to give on the subject (open-ended question). This could be something relating to any of the recommendations that we introduced or something new.

### Third round

In the final round, we presented participants with recommendations that had not reached 70% agreement (for either recommending it or not recommending it) in round two, asking for further clarification or substantiation. Additionally, we reformulated a few of the recommendations that participants had not yet agreed upon in round two and presented them again in round three. These were recommendations for which participants’ responses to the open-ended questions in round two indicated a certain nuance, complexity or controversy. For example, one of the original recommendations in round two read as ‘You can use digital contact as a way to maintain a connection with each other. Even when the digital form of contact is not immediately understood by someone, or maybe makes them a little upset’. In the third round, we changed this recommendation/question to ‘Sometimes support professionals and/or family members find it important to engage in digital social contact. Even when the digital form of contact is not immediately understood by the person with a disability, or maybe makes them a little upset. Do you have any suggestions for how to deal with this?’.

All rounds ended with an open-ended question, where participants could provide additional comments or share their rationale behind their answers on the closed-ended questions.

## Findings from the Delphi consultation

### Groups mostly making use of information on digital social contact

The group of experts as a whole indicated that support professionals (88%) and families of people with disabilities (73%) would mostly make use of information and advice on digital social contact, followed by managers (51%), specialists, like physicians or behavioural consultants (45%), service users (43%), IT professionals (24%), directors (18%) and technical services (16%).

### Main themes

The majority of participants indicated that the four main areas we presented to them in round one were all important in relation to digital social contact, making it difficult to prioritise them. The group of experts as a whole, ranked the four areas as follows, from most important to least important: 1. Service user support needs and possibilities (*mean rank* = 2.98, *range* = 1-4, *mode* = 3); 2. Reasons for and types of digital social contact (*mean rank* = 2.94), *range* = 1-4, *mode* = 4); 3. Materials and other requirements needed to enable digital social contact (*mean rank* = 2.65, *range* = 1-4, *mode* = 3); 4. Do’s and don’ts for organising and using digital social contact in sheltered care facility homes (*mean rank* = 2.42, *range* = 1-4, *mode* = 1). Separately, participants indicated that the topic of innovation was important to include, mainly concerning the tailored development of devices and applications and the collaboration between different parties.

Based on these results and participant’s additional comments and input from all three Delphi rounds, we eventually identified four main themes for structuring the recommendations:

1. Reasons for and types of digital social contact
2. Support and training needs
3. Materials and other requirements needed to enable digital social contact
4. Best practices and future developments

**Figure 1.**
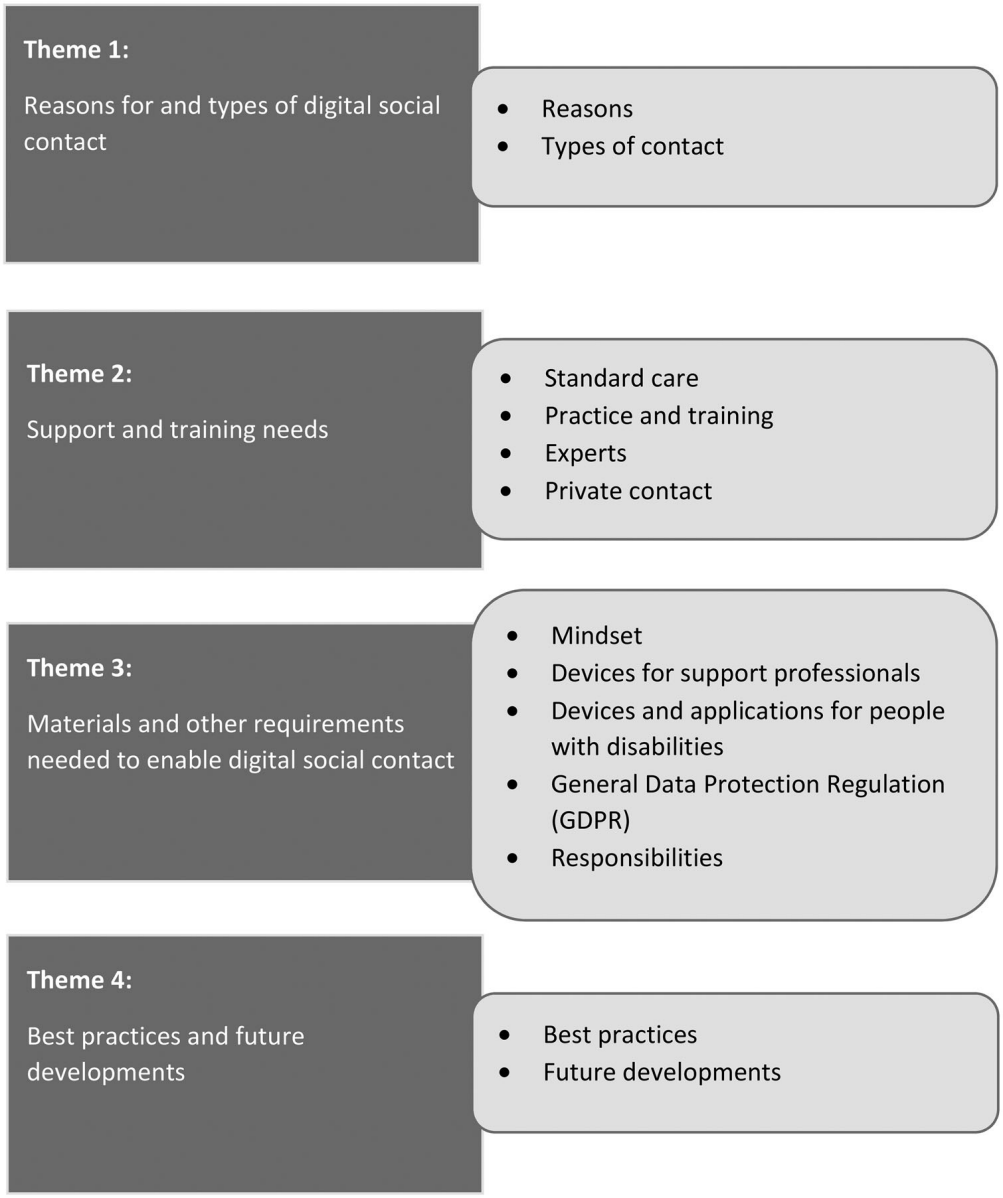
Main themes and subthemes structuring the recommendations for digital social contact.

Additionally, within each main theme, we can distinguish several subthemes, as shown in Figure 1.

The next section of this consensus statement outlines a set of specific recommendations concerning the use of digital social contact, followed by several general principles.

### Specific recommendations, and from whom action is required

Across the group of experts as a whole, the participants in our study reached a consensus regarding on the overarching recommendations concerning digital social contact for people with intellectual disabilities. This resulted in a final set of specific recommendations associated with each of the aforementioned four main themes. Table 1 shows these recommendations (arranged by the order of main themes as laid out above). Participants’ additional comments and input given in all three Delphi rounds produced supplementary context information and considerations concerning the specific recommendations, which are also shown in Table 1. Acting on the specific recommendations was seen as firstly or mainly the responsibility of sheltered care facility homes and affiliated health care organisations. However, regarding some recommendations, actions from service users, their families and direct support professionals are also required (see Table 1).

#### Differences between people with mild or moderate intellectual disabilities and people with profound intellectual and multiple disabilities

Before the results of Table 1 are shown, it should be mentioned that, overall, participants’ responses indicated no principled differentiation in the nature of the recommendations for people with mild or moderate intellectual disabilities and people with profound intellectual and multiple disabilities. All people with disabilities should at least be able to explore the options of digital social contact suitable for, and tailored to, their own particular situation. Participants in our study agreed that the feasibility and desirability of digital social contact depends highly on the individual, taking into account their abilities and disabilities, but also their digital skills and the personal preferences of both people with disabilities and their networks. Thus, in general, all recommendations and considerations pertain to all people with intellectual disabilities, on the condition that support should be individually tailored. However, participants’ responses did indicate that people with profound intellectual and multiple disabilities would face more difficulties in independently using certain types of digital contact or finding a meaningful way of digital contact than people with mild or moderate intellectual disabilities. For example, for some people singing a goodnight song together could be a very meaningful part of their day, whereas for others this might not be the case.

**Table 1.** Recommendations, from whom action is required and additional context information regarding digital social contact between people with disabilities living in sheltered care facility homes and their social networks.

Themes and recommendations	Additional context information/considerations	Requires action firstly/mainly from: • Service users (S) • Families/social networks of people with disabilities (F) • Direct support professionals (P) • Sheltered care facility homes/health care organisations (O)
**Reasons for and types of digital social contact**		
Reasons Consider that digital social contact can contribute to: – More frequent contact, which could result in a strengthening of relationships between people with disabilities and their social networks – Easier contact with family/friends not living nearby, which could result in an expansion of the social networks of people with disabilities – Continuous contact, also when someone is ill or on holiday, or when in-person contact is not possible – Participation in society of people with disabilities When exploring digital social contact as service users, family members and support professionals, try different types of digital contact to discover what is preferred and feasible for the person with a disability and their social network. Consider different devices and means, such as video calling or texting. Also contemplate a variety of aims and activities, such as having a conversation, ‘simply’ saying goodnight, playing a game, singing a song or looking through photos together	Digital social contact, in all its different forms, can have added value to a person’s well-being, particularly when used in addition to in-person contact Realise that it takes some time for people with disabilities as well as family members and support professionals to get used to digital means and activities of contact. Allow for taking small steps and repetition For many people with disabilities, some type of digital social contact appears possible. Whether digital contact is feasible and desirable depends partly on the type of disability, but also on the attitude, specific skills and expectations of both the person with a disability and their social network	S, F, P, O
Types of contact People with disabilities, family members and support professionals have a shared responsibility in deciding which type of social and digital contact is desirable and feasible		S, F, P, O
**Support and training needs**		
Standard care Integrate the possibility and support of digital social contact as part of standard care for people with disabilities		P, O
Practice and training Ensure that support professionals’ digital skills are up to date. Provide them with digital skills training as well as education on how they can explain the concept and workings of digital contact to people with disabilities (e.g. how is it possible that their family member appears on a screen in a phone) Offer all people with disabilities the opportunity to try and practice different digital means and activities of contact. Provide digital skills training for people with mild or moderate disabilities Realise the importance of digital skills of family members. Provide them with digital skills training or refer them to external possibilities	Realise that, although we live in a digital society, there are people not very familiar with the digital world and/or specific types of digital social contact. This applies to people with disabilities, but also to family members and direct support professionals Providing family members with adequate digital skills may help them to: – Take better part in digital social contact – Better support digital contact for their family member with a disability – Guide and train other family members Tips regarding the organisation of digital skills training: – Education and guidance can be offered by skilled colleagues or hired experts – Digital skills training and short courses are already available for all target groups (people with disabilities, families, direct support professionals), both offline and online – Care team meetings with support professionals are a suitable venue for discussing digital skills and applications. Family members could be included in these meetings as well	O
Experts Have digital contact experts on staff in the organisation and/or sheltered care facility home for offering advice and support to people with disabilities, family members and support professionals	The digital contact expert can be a specialised position or part of the responsibilities of a support professional	O
Private contact Allow for private contact between people with disabilities and their social networks as much as possible. The extent to which this is possible depends on the individual situation and support needs. Record specific arrangements in a person’s care plan		P, O
**Materials and other requirements needed to enable digital social contact**		
Mindset As an organisation and as support professionals, invest in cultivating an inclusive mindset towards digital developments within the sheltered care facility home. Develop a shared vision and policy on people with disabilities engaging in digital social contact		P, O
Devices for support professionals Provide support professionals with digital devices and applications they can use to share updates with family members about their relative/friend	Sometimes people with disabilities and family members ‘stayed in touch’ through support professionals and family members exchanging messages with each other The use of an application following GDPR (General Data Protection Regulation) guidelines is strongly recommended (e.g. the Dutch application Klasbord)	O
Devices and applications for people with disabilities When possible, use existing devices and applications for digital social contact, including consumer technology Look for user-friendly products, apply settings and supporting accessories to make them easier to use (e.g. larger font, speech technology, phone standard) If necessary, invest as an organisation in developing new devices and applications, or make adjustments to existing devices and applications	Important aspects to bear in mind when deciding which device or application to use: – It concerns personal contact, not work or care-related contact – Alignment with skills and preferences of people with disabilities and their social networks – Alignment with what other people generally use, i.e. regular consumer technology. This also makes it easier to provide support – Ease of use – Recommendations following the GDPR – Risks of abuse (e.g. concerning data privacy or ill-intentions of other people online) – Costs for and executability by the sheltered care facility home	P, O
General Data Protection Regulation (GDPR) As an organisation, provide a guideline on which devices and applications are suitable to use according to the GDPR		O
Responsibilities As an organisation, develop a policy on who is responsible for ensuring that the materials and other requirements for digital social contact are present. The general consensus is that service users/their families are firstly responsible for obtaining devices and applications needed for digital social contact meant for personal use. If service users/their families are not able to do so, the sheltered care facility home has a responsibility to offer practical or financial support	The general view of our participants is that service users/their families and the sheltered care facility home share a responsibility in ensuring that the materials and other requirements for digital social contact are present. However, facilities may differ in their specific regulations (e.g. whether the service user or facility is responsible for arranging an internet connection or a computer) In obtaining the necessities for digital social contact, sheltered care facility homes can offer practical support, such as giving advice or helping with the making of a purchase. Financial support can also be offered by, for example, purchasing or lending shared devices and applications for digital social contact Important aspects to consider regarding who is responsible for obtaining the necessities for digital social contact: – It concerns personal contact, not work or care-related contact – Sheltered care facility homes have a responsibility in supporting social contact (both in-person and digital contact) – If a support need (practical or financial) in obtaining devices and applications is present – If a sheltered care facility home has well-founded reasons for not allowing certain devices and applications (e.g. related to privacy, finances or technical support). Decisions about restrictions in this matter should never be made lightly, and service users and their families should be informed about them – Finding a balance between a service user’s need for autonomy (e.g. service users can decide themselves which devices and applications they want to use) and the costs for and executability by a sheltered care facility home (e.g. it is often not do-able for a sheltered care facility home to arrange for policies, technical services and practical support for an unlimited range of devices and applications)	S, F, O
**Best practices and future developments**		
Best practices Invest in sharing knowledge and ‘best practices’ between support professionals and organisations. Also invest in sharing knowledge with external parties, such as experts and developers in the field of disability care and/or health technology		O
Future developments Invest in making it easier to find what devices and applications already exist (e.g. providing an easy-access online overview or databank) Invest in inclusive design when developing devices and applications Invest in working together with other sheltered care facility homes/health care organisations and technology experts when developing devices and applications		O

#### Reasons for and types of digital social contact

Participants in our study agreed that digital social contact can have added value to the well-being of people with disabilities, particularly when used in addition to in-person contact. They agreed that digital contact can contribute to more frequent contact with one’s social network and easier contact with people not living nearby. Participants recommended that different types and means of digital contact are tried out to discover what is preferred and feasible for people with disabilities and their social networks.

#### Support and training needs

Participants in our study agreed on integrating the possibility and support of digital social contact as part of standard care for people with disabilities. Additionally, they agreed that digital skills training, tailored practice and support should be available for all involved (i.e. people with disabilities, family members, support professionals).

#### Materials and other requirements needed to enable digital social contact

Participants in our study agreed that health care organisations should invest in developing a shared vision and policy on people with disabilities engaging in digital social contact. Additionally, participants agreed on using existing, user-friendly, devices and applications for digital social contact when possible. They also advised to, if necessary, invest in adjusting products or developing new ones. The general view of our participants was that people with disabilities/their families and the sheltered care facility home share a responsibility in ensuring that the materials and other requirements for digital social contact are present.

#### Best practices and future developments

Participants in our study agreed on investing in sharing knowledge and ‘best practices’ within organisations and with external parties (e.g. developers in the field of disability care). Additionally, they agreed on investing in inclusive design and making it easier to find what devices and applications already exist.

### General principles for using digital social contact

Based on the specific recommendations and participants’ additional input given in all three Delphi rounds, we can distinguish several general principles relevant for when the use of digital social contact is considered by people with disabilities, their social networks, support professionals and managers and directors of sheltered care facility homes. These general principles apply to both people with mild or moderate (intellectual) disabilities and people with profound intellectual and multiple disabilities.

#### General principles for all parties involved (i.e. people with disabilities and their social networks, support professionals, managers and directors of sheltered care facility homes)

- We are living in a digital society; having access to digital social contact contributes to the societal participation and inclusion of people with disabilities
- For each individual with a disability and their specific circumstances, it should be considered which form of social contact (in-person or digital) is beneficial and feasible. In general, social contact with family and friends contributes to a person’s well-being and quality of life
- Providing digital social contact for people with disabilities asks for an inclusive mindset in order to use the possibilities of current technological devices and applications
- Think in terms of possibilities, not in limitations

#### General principles for people with disabilities, their social networks and direct support professionals

- When exploring digital social contact, try to vary in means and activities, corresponding with a person’s abilities
- Take time to practice and become more familiar with digital contact

#### General principles for managers and directors of sheltered care facility homes, and developers of technology

- Sheltered care facility homes have a responsibility in supporting participation and social contact for their service users, which would include both in-person and digital forms of contact
- Involve target groups (e.g. people with disabilities, families, support professionals) when developing materials and products (e.g. applications for digital contact, but also training materials)
- Invest in exchanging knowledge and best practices concerning digital social contact and other digital developments between organisations and professionals

## Discussion

Building upon findings of our previous studies (Bakkum *et al.* 2021, Bakkum *et al.* 2022) and those of others (Ramsten *et al.* 2019, Araten-Bergman and Shpigelman 2021), this study synthesises findings from literature and recent practice using a modified Delphi study design involving the input of a diverse group of experts (i.e. people with disabilities and their families, support professionals, behavioural consultants, managers and directors of sheltered care facility homes, scientists and industry experts) on the subject of digital social contact. Based on consensus between these experts, the current article provides a comprehensive outline of overarching recommendations and considerations in relation to digital social contact for both people with mild or moderate intellectual disabilities and people with profound intellectual and multiple disabilities living in sheltered care facility homes. Four main themes were identified: 1. Reasons for and types of digital social contact; 2. Support and training needs; 3. Materials and other requirements needed to enable digital social contact; and 4. Best practices and future developments. Within each theme, specific recommendations were generated. Overall, the findings of our study underpin digital social contact as a valuable additional option to in-person contact, which is in accordance with other studies (e.g. Araten-Bergman and Shpigelman 2021). Also, the findings highlight the merit of exploring different means and activities when engaging in digital social contact, tailored to people’s preferences and abilities. Participating experts believed that support professionals and families of people with intellectual disabilities would make use of the content of the recommendations formulated in this study the most. Nonetheless, the execution of many of the recommendations requires action and policy from managers and/or directors of sheltered care facility homes. Although this study concerns people with intellectual disabilities living in sheltered care facility homes, other research suggests that it is likely that the value of having various ways for communicating and staying connected to other people also extends to people with and without disabilities not living in sheltered care facility homes (Lieberman and Schroeder 2020, Spassiani *et al.* 2022, Van Asselt-Goverts *et al.* 2015). Additionally, it is relevant to realise that this study covers digital types of personal social contact (between people who know each other). Digital types of work or care-related contact (e.g. eHealth consults) may ask for different recommendations (e.g. concerning data privacy).

Participants in our study indicated that facilitating and fostering digital social contact, and considering it as part of standard care, allows people with disabilities living in sheltered care facility homes to explore and participate in the present digital world, as also indicated in previous research (Goggin *et al.* 2017, Alfredsson Ågren *et al.* 2020, Spassiani *et al.* 2022). This relates to Hanzen *et al.* (Hanzen *et al.* 2017) stating that for people with visual and severe or profound intellectual disabilities participation means active engagement and involvement in social contacts and societal activities, including opportunities for inclusion, experiences and discovery. Additionally, digital social contact could strengthen and broaden the social networks of people with disabilities, especially when used not as an alternative but as an *addition* to in-person contact (Lieberman and Schroeder 2020, Spassiani *et al.* 2022). The consensus in the current study is that, in general, the potential benefits of digital social contact pertain to both people with mild or moderate intellectual disabilities and people with profound intellectual and multiple disabilities. However, our participants agreed that it would be more difficult for people with profound intellectual and multiple disabilities to independently use certain devices or applications, or to have meaningful interactions through screens, which is in line with previous studies (Lancioni *et al.* 2013, Dyzel *et al.* 2020). In this light, it is important to consider that digital social contact may be used for a variety of activities; video calling, for example, may be used for having a conversation, but may also be used for singing a song, looking through photos or simply saying goodnight. Our findings indicate agreement on the notion that which form of digital social contact is feasible and desirable depends highly on the individual and their circumstances, and should be evaluated individually. Supported by other research (Nijs and Maes 2021, Ramsten *et al.* 2019, Parsons *et al.* 2008, Bakkum *et al.* 2022), the recommendation of our study is that the abilities, digital skills and preferences of both people with disabilities and their networks, as well as the digital skills of support professionals and the mindset and technical facilities within an organisation are taken into account. This is similar to the considerations involved with facilitating and supporting social contact for people with intellectual disabilities in general. Some people with intellectual disabilities and/or their families may decide not to use digital social contact. For example, because interacting through a screen brings them too little joy or feels too limiting, or too much technical or practical support is needed (for either the person with a disability or their family). In agreement with previous research (Barlott *et al.* 2020), the consensus in our current study is – for all people with intellectual disabilities – to at least explore multiple options of digital social contact, tailored to an individual’s situation *with personalised support*, and to structurally practice digital skills.

Supported by previous studies (Frielink *et al.* 2021, Barlott *et al.* 2020, Goggin *et al.* 2017, Araten-Bergman and Shpigelman 2021), a recommendation from our current study is to, where possible, use devices and applications that are commonly used in the general population (i.e. consumer technology), to best promote the participation of people with intellectual disabilities in the present world. With education, practice and personalised support many people with intellectual disabilities will be able to use consumer technology as is. Some studies indicate that others will need tailored adjustments to make the use of consumer technology easier (Bakkum *et al.* 2021, Dyzel *et al.* 2020). For some people, using the built-in accessibility features (e.g. larger font, speech technology) or supporting accessories (e.g. phone standard, larger screen, braille keyboard) may be enough to be able to use consumer technology. For others, more extensive adjustments or new developments may be necessary. Developing tailored products for certain subgroups of people with disabilities might be an option, if subgroups can be differentiated based on shared abilities and preferences relevant to the use of digital social contact. In general, participants in our study recommend that health care organisations invest in researching and sharing knowledge on how digital social contact can be made easier to use for people with disabilities, and what possibilities for adjustment and support already exist. Many different kinds of products and assistive technology have already been developed within the field of both consumer and healthcare technology (e.g. Boot *et al.* 2018, Dyzel *et al.* 2020, Bakkum *et al.* 2021, Drew 2021, WIPO 2021). Furthermore, it is recommended that organisations work together, as well as with technology and digital experts, in developing adjusted or new products, as also stated in previous studies (e.g. Van Delden *et al.* 2014). Collaboration generally allows for cost reductions and the development of tailored products that are more widely applicable (Littler *et al.* 1995).

Another recommendation emerging from the current study is that people with disabilities and their networks are involved in developing adjusted or new products, aiming for Universal Design. This is relevant when it concerns products specifically intended for people with disabilities, but also when it concerns products intended for the general consumer market. People with disabilities make up a significant part of the population (Woittiez *et al.* 2019, Global Burden of Disease Collaborative Network 2018, World Health Organisation (WHO) and The Worldbank 2011), and therefore should be part of the prospective end-users. Moreover, there is an upcoming movement encouraging the citizen’s perspective and public values to be an inherent part of developing technologies (Broerse and Buning De Cock 2012). Other studies state that this asks for participation from citizens and governance from governments, as technology companies are likely to have competing interests (i.e. the public good versus financial motives) (Stikker *et al.* 2020, Van Dijck *et al.* 2018). Although involving end-users, particularly people with vulnerabilities or disabilities, might be seen as complex and perhaps a costly process (Goedhart *et al.* 2021, Hoeft *et al.* 2014), studies have shown that applying participative or inclusive methods in research and development (e.g. co-creation or design thinking) increases the accessibility, use and satisfaction of products or services (Ward *et al.* 2017, Derks *et al.* 2022, Langdon *et al.* 2020, Altman *et al.* 2018, O’hern and Rindfleisch 2010, Frankena *et al.* 2016). Similar effects are visible regarding the development and implementation of policies and interventions (Johnson and May 2015). Furthermore, as our participants indicated, people with disabilities and their networks often think of creative and innovative ideas which can be of added value to developers and experts in the field.

In addition to possible needs for adjustment or support, aspects concerning data privacy should also be considered when using consumer technology. In the European Union, consumer technology and health care organisations all have to follow the General Data Protection Regulation (GDPR) (European Union n.d.). A large element of the GDPR involves informing users about which data is being collected, how data is being handled and what the possible risks are for users’ privacy. In addition, users are asked to agree to the conditions presented to them before getting access to the technology or product. However, the provided information to people is often complex, especially for people with intellectual disabilities. Therefore, extra effort should be taken to ensure that they, too, can make informed decisions on whether or not to use a particular product (Marteau *et al.* 2001). For people living in sheltered care facility homes, making decisions about which technology or product to use and ensuring informed decision-making is often a shared responsibility between people with disabilities, family members and support professionals (Godolphin 2009). Participants in the current study agreed that health care organisations should actively and repeatedly offer support and guidance for people with disabilities, their families and support professionals on how to use their rights under the GDPR (i.e. as a supportive tool in examining possibilities, not a restrictive one), and which devices and applications are suitable to use, employing existing tools (e.g. Autoriteit Persoonsgevens n.d.) if possible.

### Limitations and future directions

Participants for the Delphi consultation were recruited through convenience sampling, and may therefore not be representative of the stakeholder groups they represent. To limit selection bias, we a-priori defined a recruitment frame to maximise variation across relevant dimensions and assured an anonymous process of filling in the questionnaires. Additionally, this consensus statement was formulated during the COVID-19 pandemic with specific attention for the COVID-19 context, and thus may partly reflect concerns associated with this period. A concern might be that the answers of participants in our study with an intellectual disability were less heard or valued than the answers of other participants. However, we took several measures to lower the risk of this happening (e.g. using mostly closed-ended questions, assuring anonymity and allowing support with filling in the questionnaires, receiving input and advice from several experts-by-experience when developing the questionnaires). Although, in general, the overarching recommendations apply to both people with mild or moderate intellectual disabilities and people with profound intellectual and multiple disabilities, there might be differences in views regarding these two groups that are not examined in this study. Particularly differences between these groups and individuals concerning the specific execution of the more generally formulated recommendations. Future research could focus on how exploring that what is preferred by and works best for an individual can be stimulated and facilitated. Additionally, further research regarding tailored development of devices and applications is needed. Part of this research should also focus on how different parties (e.g. health care organisations, technology experts and end-users) can fundamentally collaborate.

## Conclusion

Digital forms of social contact are increasingly used worldwide, further accelerated by the COVID-19 pandemic. Digital social contact can contribute to the participation in society and well-being of people with intellectual disabilities, especially when used in addition to in-person contact. This modified Delphi study resulted in a consensus statement recommending to explore multiple options of digital contact, tailored to people’s abilities and preferences. Furthermore, it is recommended that end-users, developers, and other relevant stakeholders work together in further developing technology for digital social contact, be it intended for people with intellectual disabilities or the general consumer market, aiming for inclusive-by-design as the future standard to which to adhere.

## Data Availability

The data supporting the conclusions of this article are included within the article. The questionnaires used and datasets analysed during the current study are available on DataverseNL. Access can be requested through Carlo Schuengel (c.schuengel@vu.nl).
